# The genetic differentiation of a cricket (*Velarifictorus micado*) with two modes of life cycle in East Asia after the middle Pleistocene and the invasion origin of the United States of America

**DOI:** 10.1002/ece3.6967

**Published:** 2020-11-09

**Authors:** Baiqiu Wang, Kai Li, Zhu‐Qing He

**Affiliations:** ^1^ School of Life Sciences East China Normal University Shanghai China

**Keywords:** biogeography, climate shifts, diversification analyses, East Asia, invasive species, life cycle, Mid‐Pleistocene, *Velarifictorus micado*

## Abstract

The cricket *Velarifictorus micado* is widely distributed in East Asia and colonized the United States of America (the USA) in 1959. It has two life cycles: egg and nymph diapause. We aimed to investigate the biogeographic boundary between them and determine when and why *V. micado* diverged. Mitochondrial fragments including COI and CytB were used for haplotype network, demographic analysis, and divergence time estimation in individuals of East Asia. We selected several samples from the USA to find out the colonization origin. The haplotype network indicated there were three lineages based on COI, NE lineage (the egg diapause and mainly distributed in the northern regions), SE lineage (the egg diapause and mainly distributed in the southern regions), and SN lineage (the nymph diapause and mainly distributed in the southern regions). The molecular chronograms indicated that the first divergence of *V. micado* into two main lineages, NE and southern lineages (SE and SN), was essentially bounded by the Yangtze River. It occurred around ~0.79 Ma (95% HPD: 1.13–0.46 Ma) in the Middle Pleistocene Transition. This was followed by the divergence of the southern lineage into two sublineages, SE and SN lineage, occurred around ~0.50 Ma (95% HPD: 0.71–0.25 Ma), corresponding to the time of development of glaciers in various parts of the Qinghai–Tibet Plateau (QTP) (0.73–0.46 Ma). SE lineage might originate from southwestern China based on the comparison between the haplotype network based on COI and CytB. Our study suggested that divergences of lineages have twice co‐occurred with tendency of cooling climatic in Asia after the Mid‐Pleistocene, and the life‐history strategy may play an important role in lineage diversification. Additionally, our results indicated that the USA populations were revealed at least twice separate Asian invasions. These both belonged to the egg diapause, which might provide a new perspective for invasion control.

## Graphical abstract

Cricket *Velarifictorus micado* has diversified to three lineages, including the egg diapause populations lived in north Asia, the egg diapause populations lived in South Asia, and the nymph diapause populations lived in South Asia. Our study suggested that the twice divergence of *V. micado* co‐occurred with tendency of cooling climatic in Asia after the Mid‐Pleistocene.

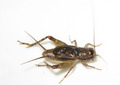

**Questions the manuscript attempts to address**

Cricket *Velarifictorus micado* is widespread in East Asia and found that it had two modes of life cycle. However, there are still two main questions unsolved.

1. Biogeography of two life cycle modes in most regions of East Asia and the origin invasion of the USA

Methods: wide sampling and amplify mitochondrial fragments for haplotype network

2. Divergence time of the lineages and important geological and climatic events that may be correlated with the evolution of lineages

Methods:

- Divergence time estimation based on COI to reveal which important events might result in lineage divergence of the *V. micado*.
- Evolutionary difference between two genes to speculate the origin of the ancient residents.

## 1 INTRODUCTION

The collision and continuous compression between the Indian plate and the Asian continent, a major event in the Phanerozoic eon, resulted in the emergence and uplift of the Himalayas and the Qinghai–Tibet Plateau (QTP) (59–50 Ma) (Hall, 2002; Hu et al., 2017; Murphy et al., 1997; Searle, 1987; Wang et al., 2014; Zahirovic et al., 2012). Geographic changes resulted in the formation and development of Asian water system, such as the modern Yangtze River established during 23 Ma (Zheng et al., 2013), and driving the climate changes in Asia. The Yangtze River, a natural barrier, isolated the species distributed in northern and southern China. The climatic oscillations occurred frequently during the Pleistocene. The cold glacial and warm interglacial cycled regularly and repeatedly. Glaciers developed gradually in various parts of the QTP after the Kunhuang Movement (0.9 Ma) (Wu et al., 1999; Yao et al., 2000; Zheng, 2000; Zhou et al., 2002). Glacial cycles had an important impact on the distribution and genetic structure of a species (Hewitt, 2000, 2004). Climate oscillation occurred in Asia during Pleistocene has affected the current distribution of species (Jia & Zhang, 2019; Ye et al., 2014; Qu et al., 2011), leading to genetic differentiation in species (Gu et al., 2013; Hazlitt et al., 2014; Rodrigues et al., 2014; Xu et al., 2009; Ye et al., 2018) and speciation in Asia (Bao et al., 2010; DeChaine et al., 2014; Lin et al., 2016; Shih et al., 2011).

Individuals colonized to new habitat due to the environmental changes in original habitat or population expansion. New behavioral, physiological, and genetic changes occurred in the adaption to new habitat (Avise et al., 1998). Studies suggested that life history is related to environmental factors (Marten et al., 2006; Papadopoulou et al., 2009). For example, the number of generations each year of *Locusta migratoria* (Orthoptera: Acrididae) increased with the decrease of latitudes in China (Chen, 1999; Guo et al., 1991; Tanaka & Zhu, 2008). The different life history within species played a more important role in speciation than geographic isolation, as in *Teleogryllus commodus* distributed in Southeastern and Northeastern Australia (Hogan, 1967), *Pternemobius taprobanensis* and *Pternemobius fascipes* distributed from the tropical to temperate region in East Asia (Masaki, 1983). Genetic data also indicated that individuals with different life cycles had been differentiated (Harrison & Bogdanowicz, 1995; Snyder et al., 2009).

*Velarifictorus micado* (Saussure, 1877) is widely distributed in East Asia, including China, Russia, Japan, Korea, Cambodia, Vietnam, Indonesia, and nearby islands, covering both the Palearctic and Oriental realms. The distribution range of this species has gradually expanded since its introduction to the United States in 1959 (Alexander & Walker, 1962; Bowles, 2018). It was also found that this cricket has two modes of life cycle. In a population that diapause as eggs, adults sing from August to October, and their eggs hatch after getting through the winter. In the other population that diapause as nymphs, adults sing in May–July hatch quickly after mating and get through the winter as nymphs. Different adaptation types of *V. micado* lead to differentiation of reproductive modes and might result in speciation among populations due to different life cycle patterns (He & Takeda, 2013). However, it is unknown that distribution characteristics of egg diapause and nymph diapause populations in Asia. The association among climatic fluctuations in Asia, life cycle and lineage divergence of *V. micado* remain poorly understood. We propose here that, (a) distribution of egg diapause and nymph diapause populations in China is basically isolated by a great geographic barrier, such as the Yangtze River, and the type of life cycle in America might be egg‐diapausing due to the similar latitude to northern China. (b) Geographic climate fluctuations in the Pleistocene of Asia might drive the diversification of life‐history strategy, which affected the genetic differentiation of *V. micado*. Accordingly, our research will use the cricket that widely distributed in temperate and tropical as the material. Aimed to find out suitable gene markers which can distinguish the two groups and the biogeographic boundary between them, investigate when and what drive *V. micado* diversification and the invasion origin of the USA, mitochondrial fragments COI and CytB were extracted from the large sampling specimens and used for phylogenetic analysis, time estimation, and demographic analysis.

## 2 MATERIALS AND METHODS

### 2.1 Sample collection and genomic DNA extraction

**FIGURE 1:**
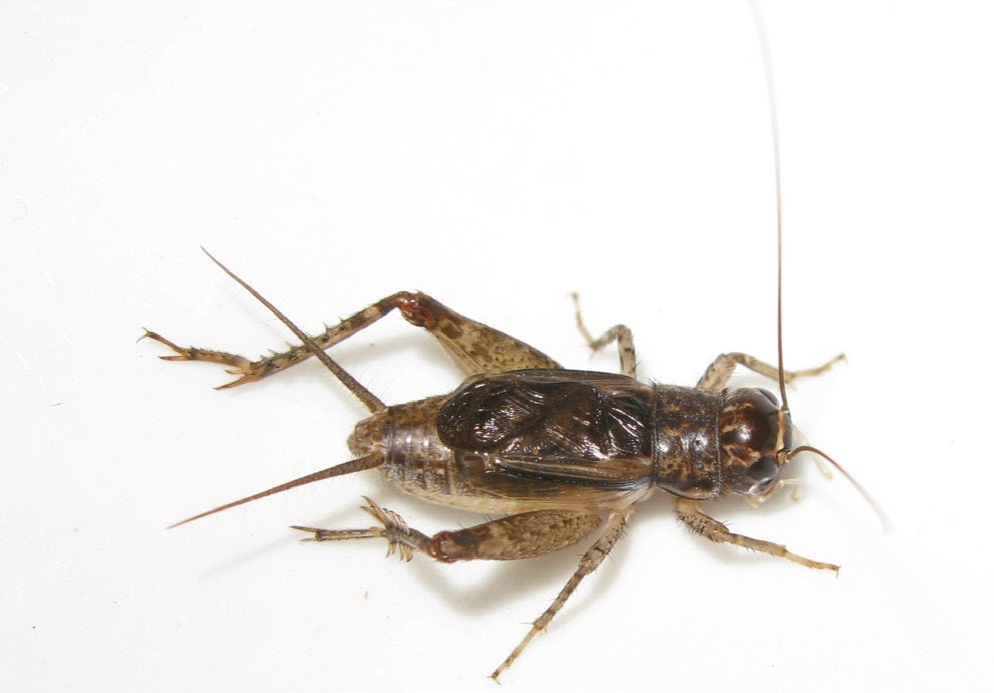
The picture of *Velarifictorus micado* taken by Zhuqing He

**FIGURE 2:**
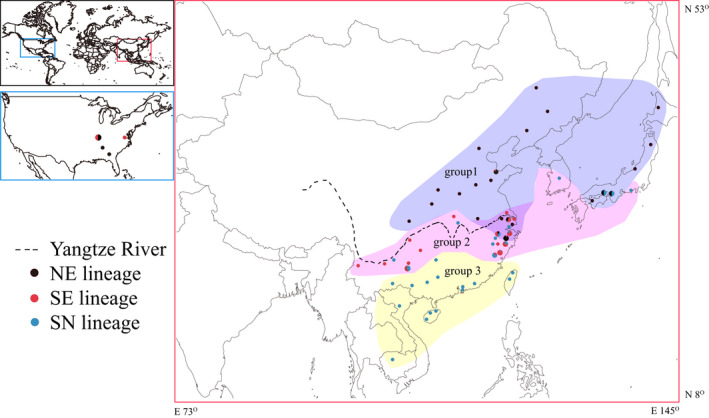
Sampling localities of *Velarifictorus micado*. Black, red, and blue circles represent the NE (the individuals that produced with egg diapause and mainly distributed in the northern regions), SE (the individuals with egg diapause and mainly distributed in the southern regions), and SN (individuals that produced with nymph diapause and mainly distributed in the southern regions) lineages based on COI, respectively. Circle sizes are proportional to the number of individuals. The colorful regions represent the groups (1, 2, and 3) defined by SAMOVA

A total of 346 individuals from 70 *V. micado* populations were collected, 55 populations from China, 8 from Japan, 1 from Korea, 1 from Vietnam, 1 from Cambodia, and 4 from the USA (Figure 1 and Figure 2). All materials were presented in 100% ethanol and stored in a freezer at −20°C, and genomic DNA was extracted from legs of the cricket using AxyPrep™ Multisource Genomic DNA Miniprep kit.

### 2.2 PCR amplification and sequencing

Universal primers for cytochrome c oxidase unit 1 (COI) and cytochrome b (CytB) genes were designed in the previous study (Table 1). The PCR procedure for the two genes included an initial denaturation at 94°C for 4 min, followed by 35 cycles of 30 s at 94°C, 30 s at 45°C and 30 s at 72°C, ending with a final extension at 72°C for 5 min. Sequencing was performed on 3730*xl* DNA Analyzer, and sequencing was proofread in ATGC Ver 7.0.2 (Genetyx Corporation) and aligned in MEGA V. 7.0.14 (Kumar et al., 2016).

**TABLE 1:** Primer sequences used in this study

Gene region	Primer name	Sequence (5′–3′)	
COI	LCO	GGTCAACAAATCATAAAGATATTGG	Simon et al. (1994)
	HCO	TAAACTTTCAGGGTGACCAAAAAATCA	
Cytb	REVCB2H	TGAGGACAAATATCATTTTGAGGW	Simmons and Weller (2001)
	REVCBJ	ACTGGTCGAGCTCCAATTCATGT	

### 2.3 Genetic analysis

#### 2.3.1 Genetic polymorphism

Base substitution saturation test was performed in DAMBE (Xia & Xie, 2001) for phylogenetic analysis. Different haplotype, haplotype diversity (Hd), and nucleotide diversity (π) were calculated in DNASP 5.10.01 (Librado & Rozas, 2009). Defining groups of populations that were geographically homogeneous and maximally differentiated from each other was implemented in SAMOVA 1.0 (Dupanloup et al., 2002). Processes for K from 2 to 10 were running using 100 simulated annealing approach. A median‐joining (MJ) haplotype network to test the relationships between groups was constructed in POPART (Leigh & Bryant, 2015), and each group has its own color. Genetic differentiation (*F_ST_*) among different lineages and the analysis of molecular variance (AMOVA) were calculated in ARLEQUIN 3.5.2.2 (Excoffier & Lischer, 2010). *F_ST_* was performed with pairwise difference genetic distance. AMOVA was used for test genetic structure and genetic variation analysis. Statistical significance of the estimates was assessed by 10,000 permutations.

#### 2.3.2 Historical demographic changes

Neutrality tests were used to speculate historical demographic change and implemented in ARLEQUIN 3.5.2 (Excoffier & Lischer, 2010), including Tajima’ D (Tajima, 1989) and Fu’ Fs (Fu, 1997) with 1,000 simulated samples. These statistics assume that the population has a constant size at mutation‐drift equilibrium. A significantly negative value indicates there are excess of low frequency haplotypes in the population, suggesting that there might be expansion. Moreover, the value of Tajima’ D is negatively correlated with the time since introduction. Namely, the higher (positive) the value of Tajima’ D, the more recent introduction, indicating that these populations are recovering from the bottleneck effect.

#### 2.3.3 Divergence time estimation

Estimating divergence time was based on mitochondrial markers of COI in BEAST 2.5.0 (Bouckaert et al., 2014). Best substitution model was evaluated using Bayesian information criterion (BIC) selected in the program JModeltest v.2 (Darriba et al., 2012). A set of 15 specimens comprising 6 other *Velarifictorus* species and 9 ancient haplotypes of *V. micado* was employed for the estimation of divergence time (Table 2). A relaxed clock log normal was applied with 1.7% per site per lineage per million years for COI (Allegrucci et al., 2011, 2017; Chobanov et al., 2016; Kaya & Çiplak, 2016; Kiyoshi & Sota, 2006; Papadopoulou et al., 2010; Pons & Vogler, 2005; Shapiro et al., 2006). Divergence time was estimated using a Yule model. After running the chains for 500 million generations in BEAST, the stability on the log‐likelihood curves and the split frequencies were checked in Tracer v1.7.1. The parameter estimates with ESS > 200 were accepted. Discarding the first 20% as burn‐in and summarize trees in Tree Annotator v2.5.0. Trees were visualized with the FigTree v1.1.2 (These software are included in the BEAST package).

**TABLE 2:** Geographic information and GenBank accession number of the genus *Velarifictorus*

Species (population)	Coordinates		GenBank No.		Reference
	Lat.	Long.	COI	CytB	
*Velarifictorus_dianxiensis*	N24°44′58.9″	E97°33′45.54″	MH992037		Chen et al. (2018)
*Velarifictorus_beybienkoi*	N37°25′58.84″	E118°40′8.54″	MH992030		Chen et al. (2018)
*Velarifictorus_aspersus*	N27°21′16.49″	E119°55′57.38″	MH992029		Chen et al. (2018)
*Velarifictorus_flavifrons*	N21°55′37.21″	E101°15′22.31″	MH992033		Chen et al. (2018)
*Velarifictorus_ornatus*	N25°37′19.37″	E110°25′32.11″	MH992024		Chen et al. (2018)
*Velarifictorus_agitatus*	N22°09′4.00″	E100°41′29.04″	MT998290		This study
*Velarifictorus_micado*					
HLJ	N46°02′57.67″	E125°57′22.18″	MT997308318	MT996525–MT996536	This study
LN	N41°40′30.64″	E123°27′30.98″	MT997319		This study
JL	N43°50′45.16″	E126°33′35.93″	MT997320–MT997321		This study
BJ	N39°54′11.46″	E116°24′2.71″	MT997322		This study
HNJY	N35°04′42.16″	E112°42′26.30″	MT997323–MT997344	MT996537‐MT996554	This study
HNPY	N35°51′7.02″	E115°29′53.63″	MT997345	MT996555	This study
SXXA	N34°20′33.29″	E108°56′7.28″	MT997346		This study
SXNS	N33°18′44.97″	E108°18′33.70″	MT997347		This study
SCCD	N30°39′8.18″	E104°04′20.98″	MT997348		This study
SDDY	N37°26′53.41″	E118°34′35.12″	MT997349–MT997362	MT996556–MT996568	This study
SDTA	N36°0′3.16″	E116°43′12.42″	MT997363–MT997378	MT996569–MT996583	This study
HBXY	N32°0′39.61″	E112°06′57.21″	MT997379–MT997382	MT996584–MT996587	This study
HBWH	N32°0′39.61″	E112°06′57.21″	MT997383–MT997384	MT996588	This study
AHHF	N31°49′20.30″	E117°13′17.39″	MT997385	MT996589–MT996590	This study
JSNJ	N32°03′57.43″	E118°46′56.02″	MT997386–MT997387		This study
JSYZ	N32°23′46.68″	E119°24′27.01″	MT997388		This study
JSZJ	N32°11′25.78″	E119°25′6.73″	MT997389		This study
GZXY	N25°05′41.85″	E104°53′36.79″	MT997390–MT997399	MT996591–MT996601	This study
GZGY	N26°35′33.05″	E106°43′7.60″	MT997400	MT996602	This study
GZRH	N27°50′48.13″	E106°20′51.17″	MT997401–MT997402	MT996603–MT996604	This study
GZZY	N28°35′24.74″	E107°35′55.73″	MT997403	MT996605	This study
GXLG	N25°15′3.64″	E110°11′20.83″	MT997404		This study
GXPM	N22°56′40.47″	E105°59′35.62″	MT997405–MT997411	MT996606–MT996612	This study
GXSL	N23°36′42.50″	E108°33′25.47″	MT997412–MT997414	MT996613–MT996615	This study
GXJX	N23°04′14.23″	E106°27′20.61″	MT997415–MT997418	MT996616–MT996622	This study
ZJJD	N29°28′38.51″	E119°16′33.40″	MT997419		This study
ZJTT	N30°08′9.03″	E119°01′14.75″	MT997420–MT997437	MT996623–MT996636	This study
ZJFY	N27°53′28.88″	E119°10′15.09″	MT997438	MT996637	This study
ZJBMS	N28°38′38.96″	E119°09′0.31″	MT997439–MT997440		This study
ZJTM	N30°21′0.63″	E119°25′28.82″	MT997441–MT997450	MT996638–MT996642	This study
ZJBSZ	N27°45′16.60″	E119°12′21.25″	MT997451–MT997476	MT996643–MT996666	This study
ZJQY	N27°37′20.59″	E119°03′28.56″	MT997477		This study
ZJGT	N29°15′29.68″	E118°08′59.26″	MT997478–MT997481	MT996667–MT996670	This study
ZJWYL	N27°42′50.78″	E119°39′29.97″	MT997482–MT997485	MT996671–MT996676	This study
ZJDP	N30°0′7.75″	E120°04′8.21″	MT997486–MT997526	MT996677–MT996717	This study
ZJSX	N30°01′57.90″	E120°34′29.75″	MT997527–MT997544	MT996718–MT996735	This study
ZJJL	N30°35′49.36″	E121°05′46.26″	MT997545–MT997553		This study
SHCJ	N30°47′54.27″	E121°24′6.62″	MT997554		This study
SHCM	N31°37′28.83″	E121°23′33.41″	MT997555		This study
SHJS	N30°43′2.21″	E121°19′33.99″	MT997556		This study
SHMH	N31°01′58.20″	E121°26′59.25″	MT997557–MT997559	MT996736–MT996738	This study
SHBS	N31°24′25.97″	E121°29′5.70″	MT997560–MT997583	MT996739–MT996760	This study
YNKM	N24°52′58.97″	E102°49′53.66″	MT997584–MT997611	MT996761–MT996786	This study
YNMK	N25°26′40.25″	E98°51′38.72″	MT997612–MT997615	MT996787	This study
YNHK	N22°30′26.16″	E103°57′48.80″	MT997616	MT996788	This study
SZ	N22°32′43.85″	E114°03′10.40″	MT997617–MT997618		This study
FJWY	N27°52′27.58″	E117°51′22.43″	MT997619–MT997621		This study
GDHY	N22°47′28.70″	E114°27′7.57″	MT997622		This study
HNTGL	N19°39′15.29″	E111°01′15.55″	MT997623–MT997624		This study
HNJFL	N18°42′57.91″	E108°52′18.65″	MT997625–MT997629	MT996789–MT996792	This study
HNWZ	N18°46′37.03″	E109°30′46.33″	MT997630	MT996793	This study
HNXA	N19°40′50.21″	E110°21′52.01″	MT997631	MT996794–MT996796	This study
HNCJ	N19°17′59.51″	E109°03′5.28″	MT997632	MT996797–MT996799	This study
TWNT	N23°55′10.63″	E120°40′12.03″	MT997633		This study
TWWL	N24°52′21.08″	E121°32′51.11″	MT997634		This study
KR	N35°52′17.17″	E128°36′5.20″	MT997635		This study
JPSH	N34°39′4.63″	E135°10′32.81″	MT997636–MT997637		This study
JPJG	N34°58′37.63″	E138°22′59.11″	MT997638		This study
JPDB	N34°41′37.46″	E135°30′7.79″	MT997639–MT997640		This study
JPBHD	N43°13′13.18″	E142°51′48.51″	MT997641		This study
JPBK	N35°07′44.02″	E 134°29′1.11″	MT997642		This study
JPQS	N40°49′19.46″	E140°44′50.51″	MT997643		This study
JPXX	N37°55′36.72″	E139°20′22.56″	MT997644		This study
JPSS	N33°50′20.97″	E132°45′56.07″	MT997645	MT996800	This study
VN	N21°02′36.57″	E105°51′51.33″	MT997646		This study
KH	N12°43′33.90″	E104°45′56.25″	MT997647–MT997648		This study
USAGA	N33°74′83.04″	W84°39′11.13″	MT997649		This study
USATN	N37°09′37.14″	W86°18′30.94″	MT997650		This study
USAMO	N38°32′53.40″	W90°21′5.63″	MT997651–MT997652		This study
USAVA	N37°75′88.48″	W77°47′93.41″	MT997653	MT996801	This study

## 3 RESULTS

### 3.1 Genetic polymorphism and haplotype network

**FIGURE 3:**
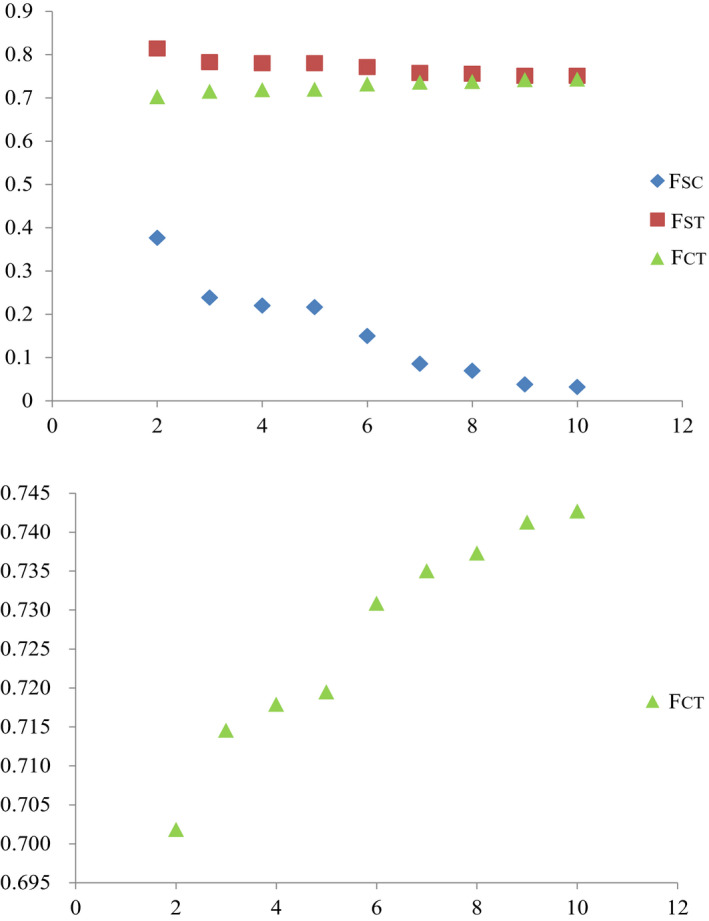
Values of fixtion indices (*F*) of different number of groups (K) based on COI. *F_CT_* is the differentiation between groups, *F_ST_* is the differentiation between populations among groups, and *F_SC_* is the differentiation between populations within groups

**FIGURE 4:**
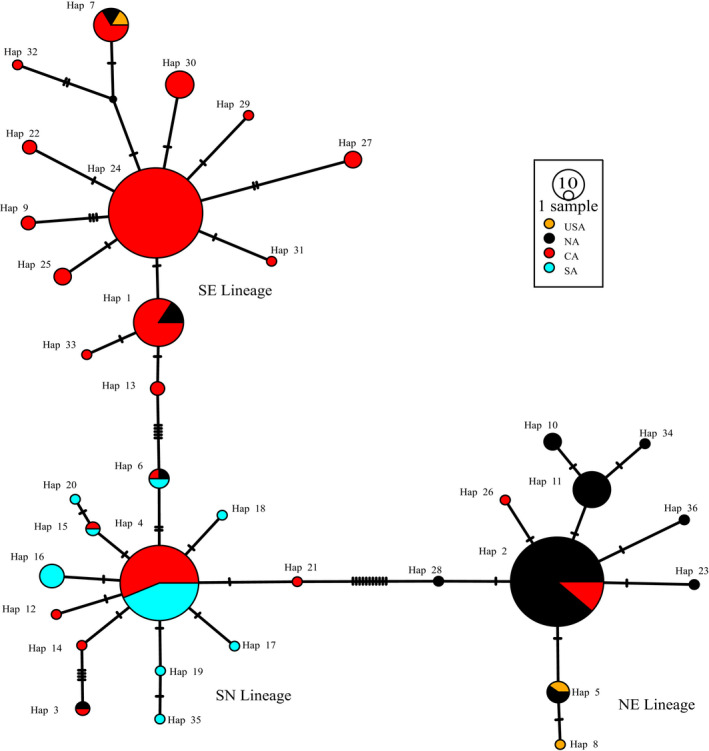
Median‐joining haplotype network based on COI constructed using DNASP and POPART. NE lineage represented the individuals that produced with egg diapause and mainly distributed in the northern regions, SE lineage represented the individuals with egg diapause and mainly distributed in the southern regions, and SN lineage represented individuals that produced with nymph diapause and mainly distributed in the southern regions. Circle size represented the number of the haplotypes, and different colors represented the different groups

COI gene (658 bp) was successfully obtained from 346 individuals. The variable sites included 12 singleton variable sites and 33 parsimony information sites. Defining groups of populations did not include those in the USA, because individuals distributed in USA were introduced since 1959 (Alexander & Walker, 1962). All populations in USA were integrated into the group USA. For the native populations, the value of *F_CT_* increased continuously from *k* = 2 to *k* = 10, and a distinct increase from 2 to 3 and from 5 to 6 using SAMOVA (Figure 3). The group comprising only one population when *k* = 6, thus, *k* = 3 was chose to defined the groups of populations. The three groups corresponded chiefly to three geographic regions. The group 1 contained populations mainly in northern China and Japan. The group 2 contained populations mainly in central China, Korea, and Japan. The group 3 was consisted of populations from southern China, Vietnam (VN), and Cambodia (KH) (Figure 2 and Table 3). The partitioning of total genetic variation in three groups using AMOVA indicated 71.45% diversity among groups, 21.76% within populations, and 6.79% among populations within groups (Table 4). Thirty‐six unique haplotypes were derived from all 346 individuals. The distribution of different haplotypes based on COI was showed in Figure 4. The haplotype distributions were divided three starry shapes in the network, NE, SE, and SN, respectively. NE contained the individuals that produced with egg diapause and mainly distributed in the northern regions. SE included the populations with egg diapause and mainly distributed in the southern regions. SN was consisted with individuals that produced with nymph diapause and mainly distributed in the southern regions (Figure 2 and Table 3). There was no sharing haplotype among the three lineages. Hap24, Hap 2, Hap 4, and Hap 11 haplotypes were the most frequent haplotypes, characterizing 25.72%, 25.14%, 17.92%, and 4.05% individuals, respectively. Hap 2 and Hap 11 were the ancestral haplotypes of NE, and Hap11 was disjoint from Hap 2, which suggested differentiation due to the long distance. Hap 4 and Hap 24 were the ancestral haplotypes of SE and SN, respectively. The partition of total genetic variation in three clades using AMOVA indicated 93.23% diversity among groups, 2.78% within populations, and 4.00% among populations within groups (Table 5). All these suggested that *V. micado* significantly differentiated between different mode of life cycles and between geographic population of northern and southern regions. Pairwise *F_ST_* among SE, NE, and SN groups ranged from 0.86294 to 0.92469 (*p* < 0.001, Table 6), which suggested that the three groups were differentiated significantly. Isolations between SE, SN, and NE group were consistent with results of AMOVA. There were Hap 5, Hap 7, and Hap 8 in USA group. Hap 5 was shared by JPSH, JPDB, and JPSS (Japan) and related to Hap 8. Hap 8 has not found in Japan. Hap 7 was shared by ZJTM, SHBS, and SDDY (Zhejiang, Shanghai, and Shandong provinces).

**TABLE 3:** Genetic information, sampling time, and phase of 72 populations

Population	Lineages	Sample size	Sampling time	Phase	hap (COI)	Tajima's D	Fu' Fs	Hd	π
Group 1						−1.52274*	−0.76144		
HLJ	NE	11	18‐viii‐2019	Adult	h 11	0.00000	0.00000	0.00000	0.00000
LN	NE	1	22‐viii‐2011	Adult	h11				
JL	NE	2	12‐vii‐2016	Adult	h11, h 34				
BJ	NE	1	9‐vii‐2015	Adult	h 2				
HNJY	NE	22	15‐viii‐2019	Adult	h 2(21), h 28	−1.16240	−0.95676	0.09100	0.00014
HNPY	NE	1	3‐x‐2019	Adult	h 2				
SXXA	NE	1	vii‐2017	Nymph	h 36				
SXNS	NE	1	15‐viii‐2010	Adult	h 2				
SCCD	NE	1	23‐viii‐2011	Adult	h 2				
SDDY	NE, SE	14	25‐vii‐2019	Adult	h 1, h 2(11), h 7, h 23(1)	−1.00845	3.81235	0.39600	0.00688
SDTA	NE	16	15‐viii‐2019	Adult	h 2	0.00000	0.00000	0.00000	0.00000
AHHF	NE	1	1‐x‐2019	Adult	h 2				
JSNJ	NE	2			h 2				
ZJSX	NE, SE	18	1‐x‐2019	Adult	h 1, h 2	−2.34276***	4.42711	0.11100	0.00253
SHCJ	NE	1	17‐viii‐2015	Adult	h 2				
SHJS	NE	1	1‐x‐2017	Adult	h 2				
SHMH	NE	3	ix‐2019	Adult	h 2				
JPSH	NE, SN	2	10‐x‐2010	Adult	h 3, h 5				
JPDB	NE, SN	2	20‐vi‐2011	Adult	h 5, h 6				
JPBHD	NE	1	30‐viii‐2011	Adult	h 10				
JPBK	NE	1	9‐viii‐2012	Adult	h 11				
JPQS	NE	1	1‐ix‐2012	Adult	h 10				
JPXX	NE	1	1‐ix‐2012	Adult	h 10				
JPSS	NE	1			h 5				
Group 2						−0.80738	−1.95584		
HBXY	SE	4	25‐ix‐2019	Adult	h 24				
HBWH	SN	2	4‐viii‐2019	Adult	h 4				
JSYZ	SE	1	23‐viii‐2011	Adult	h 1				
JSZJ	SE	1	23‐viii‐2011	Adult	h 1				
ZJTT	SE, SN	18	ix‐2018	Adult	h 1(9), h 4(5), h14 (1), h 13(2), h 21 (1)	1.12995	2.15356	0.69300	0.00524
ZJBMS	SE	2	9‐ix‐2017	Adult	h 24				
ZJTM	SE, SN, NE	10	16‐viii‐2019; 1‐vi‐2014	Nymph; Adult	h 1(3), h 2,, h 4(4), h 7(2)	−0.34421	3.40637	0.77800	0.00988
ZJWYL	SE, SN	4	3‐x‐2019	Adult	h 4, h 29				
ZJDP	SE, SN	41	4‐x‐2019	Adult	h 1(1), h 4(8),, h 15(1), h 24(20), h 30(8), h 31, h32, h33	−0.14185	0.84479	0.70000	0.00475
ZJJL	SN	9	22‐v‐2019	Adult	h 4(8), h 12				
SHCM	SE	1	19‐ix‐2017	Adult	h 1				
SHBS	SE, NE	24	25‐vii‐2019	Adult	h 1(5), h 2(9), h 7(6), h 22(2), h 24(1), h 26(1)	2.56489	6.88573	0.77500	0.01387
SZ	SE	2	21‐viii‐2011	Adult	h 9				
KR	SN	1	12‐viii‐2011	Adult	h 3				
JPJG	SN	1	12‐vi‐2012	Adult	h 6				
GZXY	SE, SN	10	25‐vii‐2019	Adult	h 4(4), h 24(3), h 25(3)	2.09337	4.21833	0.73300	0.00638
GZGY	SE	1	8‐x‐2019	Adult	h 24				
GZRH	SE	2	2‐x‐2019	Adult	h 24				
GZZY	SE	1	9‐viii‐2019	Adult	h 24				
ZJFY	SE	1	2‐x‐2019	Adult	h 24				
ZJBSZ	SE, SN	26	2‐x‐2019	Adult	h 4, h 24(25)	0.00000	0.00000	0.00000	0.00000
YNKM	SE	28	18‐viii‐2019	Nymph	h 24	0.00000	0.00000	0.00000	0.00000
YNMK	SE	4	20‐viii‐2019	Nymph	h 24, h 27(3)				
Group 3						−1.75818	−5.53709		
GXLG	SN	1	25‐vii‐2011	Nymph	h 4				
ZJJD	SN	1	17‐vii‐2018	Adult	h 4				
ZJQY	SN	1	25‐viii‐2011	Adult	h 4				
ZJGT	SN	4	3‐x‐2018	Nymph	h 4(3), h 15(1)				
FJWY	SN	3	22‐viii‐2010	Adult	h 4				
GDHY	SN	1	10‐x‐2016	Adult	h 4				
HNTGL	SN	2	11‐viii‐2018	Nymph	h 4				
TWNT	SN	1	10‐x‐2012	Nymph	h 6				
TWWL	SN	1	10‐x‐2012	Nymph	h 6				
VN	SN	1	1‐vi‐2011	Adult	h 4				
KH	SN	2	1‐i‐2019	Adult	h 4				
GXPM	SN	7	20‐iv‐2019	Adult	h 4(5), h 17(1), h 18(1)				
GXSL	SN	3	18‐iv‐2019	Adult	h 4(1), h 19(1), h 20(1)				
GXJX	SN	4	19‐iv‐2019	Adult	h 4(4)				
YNHK	SN	1	19‐viii‐2019	Nymph	h 4				
HNJFL	SN	5	iii‐2019	Nymph	h 4(1), h 16(4)				
HNWZ	SN	1	iii‐2019	Adult	h 4				
HNXA	SN	1	iii‐2019	Adult	h 16				
HNCJ	SN	1	iii‐2019	Adult	h 16				
USA						1.69413	4.19378		
USAGA	NE	1	28‐viii‐2009	Adult	h 5				
USATN	NE	1	1‐ix‐2009	Adult	h 5				
USAMO	SE, NE	2	15‐viii‐2009	Adult	h 7, h 8				
USAVA	NE	1	viii‐2009	Adult	h 7				

Note

Genetic information included lineages, neutrality tests, and nucleotide polymorphism. Genetic information was based on COI. NE lineage was consisted of the individuals that produced with egg diapause and mainly distributed in the northern regions, SE lineage was consisted of the individuals with egg diapause and mainly distributed in the southern regions, and SN lineage was consisted of individuals that produced with nymph diapause and mainly distributed in the southern regions. Hap, haplotype distribution; Hd, haplotype diversity; π, nucleotide diversity; The bold black font represents the groups (1, 2, 3, and USA) divided by SAMOVA.

* *p* < 0.05.

** *p* < 0.01.

**TABLE 4:** AMOVA in *Velarifictorus micado* categorized by SAMOVA and corresponding fixation indices based on COI marker

Source of variation	*df*	SS	VC	%V	*F*	*p*
Among groups	2	890.059	4.45636	71.45	*F*_CT_ = 0.71452	0.0000
Among populations within groups	64	220.497	0.42334	6.79	*F*_SC_ = 0.23777	0.0000
Within populations	289	392.212	1.35713	21.76	*F*_ST_ = 0.78240	0.0000

Abbreviations: %V, percentage of variation; *df*, Degree of freedom; F, multilocus *F*‐statistic; *F_CT_*, Variation among groups; *F_SC_*, Variation among populations within groups; *F_ST_*, Variation within populations; SS, sum of squares; VC, variance components

**TABLE 5:** AMOVA in *Velarifictorus micado* categorized by different lineages (NE, SE, and SN) and corresponding fixation indices based on COI marker

Source of variation	*df*	SS	VC	%V	*F*	*p*
Among groups	2	1,329.952	5.88524	93.23	*F*_CT_ = 0.93227	0.0000
Among populations within groups	82	92.395	0.25222	4.00	*F*_SC_ = 0.58988	0.0000
Within populations	261	45.768	0.17536	2.78	*F*_ST_ = 0.97222	0.0000

Note

NE lineage was consisted of the individuals that produced with egg diapause and mainly distributed in the northern regions, SE lineage was consisted of the individuals with egg diapause and mainly distributed in the southern regions, and SN lineage was consisted of individuals that produced with nymph diapause and mainly distributed in the southern regions.

Abbreviations: %V, percentage of variation; *df*, Degree of freedom; F, multilocus *F*‐statistic; *F_CT_*, Variation among groups; *F_SC_*, Variation among populations within groups; *F_ST_*, Variation within populations; SS, sum of squares; VC, variance components.

**TABLE 6:** Pairwise *F_ST_* values for the three lineages (NE, SE, and SN) of *Velarifictorus micado* based on COI

	NE	SE	SN
NE	0.00000		
SE	0.92469***	0.00000	
SN	0.91630***	0.86294***	0.00000

Note

NE lineage was consisted of the individuals that produced with egg diapause and mainly distributed in the northern regions, SE lineage was consisted of the individuals with egg diapause and mainly distributed in the southern regions, and SN lineage was consisted of individuals that produced with nymph diapause and mainly distributed in the southern regions.

* *p* < 0.05.

** *p* < 0.01.

*** *p* < 0.001.

**FIGURE 5:**
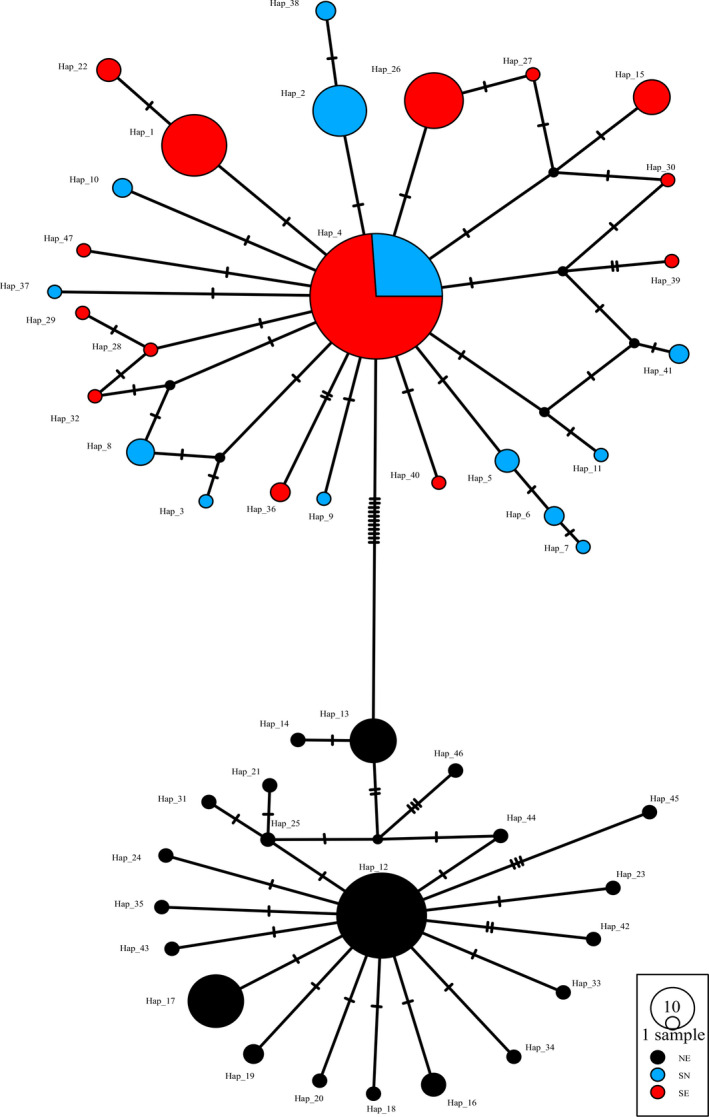
Median‐joining haplotype network based on CytB constructed using DNASP and POPART. Circle size represented the number of the haplotypes. Different colors represented three lineages (NE, SE, and SN) based on COI

Based on the CytB gene, 579 bp fragments were successfully obtained from 277 individuals. The variable sites included 22 singleton variable sites and 37 parsimony information sites. 47 unique haplotypes were derived, dividing all these samples into three groups (NE, SE, and SN) according to three main lineages of COI, all individuals were divided into two lineages significantly. Hap 12 and Hap 13 were the ancestral haplotypes of northern China (NC lineage), and Hap 4 was the ancient haplotypes of southern China (SC lineage). Coincidentally, all the individuals in NC lineage were the members of the NE, and the SC lineage was in the SE and SN (Figure 5). Based on haplotype network of CytB, southern individuals have not been diverged yet. Hap 4 was the ancient haplotype of populations with egg and nymph diapause. Individuals produced with nymph diapause in Zhejiang, Hainan, and Guangxi (ZJTT, HNBS, HNJFL, HNWZ, HNCJ, HNXA, GXPM, GXSL, and GXJX), whereas the egg and nymph diapause distributed in Yunnan, Guizhou, and Zhejiang provinces (YNKM, YNMK, GZXY, GZGY, GZRH, GZZY, ZJFY, and ZJBSZ), and the haplotype in Zhejiang was single, which suggested the ancient haplotypes of SE lineage distributed Yunnan and Guizhou (Figure 5, Table 3 and Table 9). The AMOVA results showed that the source of variation among groups categorized by three clades based on COI accounts for 81.25% (Table 7).

**TABLE 7:** AMOVA in *Velarifictorus micado* based on CytB categorized three clades (NE, SE, and SN) according COI and corresponding fixation indices based on CytB marker

Source of variation	*df*	SS	VC	%V	*F*	*p*
Among groups	4	746.238	4.89495	81.25	*F*_CT_ = 0.81249	0.0000
Among populations within groups	28	47.43	0.08838	1.47	*F*_SC_ = 0.07823	0.0000
Within populations	243	253.039	1.04131	17.28	*F*_ST_ = 0.82716	0.0000

Note

NE lineage was consisted of the individuals that produced with egg diapause and mainly distributed in the northern regions, SE lineage was consisted of the individuals with egg diapause and mainly distributed in the southern regions, and SN lineage was consisted of individuals that produced with nymph diapause and mainly distributed in the southern regions.

Abbreviations: %V, percentage of variation; *df*, Degree of freedom; F, multilocus *F*‐statistic; *F_CT_*, Variation among groups; *F_SC_*, Variation among populations within groups; *F_ST_*, Variation within populations; SS, sum of squares; VC, variance components.

### 3.2 Historical demographic changes

Based on COI, the negative and significant values of Tajima’ D and Fu’ Fs indicated past population expansion of SE and SN lineages. The negative and significant value of D also implied the expansion in the past and the stable population size of NE lineage (Table 8). However, the value of Hd (0.443–0.608), as well as the low value of π (0.00127–0.00197), implied a lower genetic diversity. The values of Tajima’ D and Fu’ Fs were positive and not significant, and there were two lineages in ZJTT, SHBS, GXXY, and USA, but it is possible that expansions recently causing the sympatric coexistence of lineages. Besides the values of Fu’ Fs were positive in ZJDP, ZJTM, SDDY, and ZJSX, there were at least two lineages in these populations.

**TABLE 8:** Neutral test of three lineages (NE, SE, and SN) based on COI

	Tajima' D	Fu' Fs	Hd	π
NE	−2.0712***	−4.2748	0.443	0.00197
SE	−1.9776**	−7.2494*	0.608	0.00160
SN	−2.1162**	−9.7367***	0.451	0.00127

Note

NE lineage was consisted of the individuals that produced with egg diapause and mainly distributed in the northern regions, SE lineage was consisted of the individuals with egg diapause and mainly distributed in the southern regions, and SN lineage was consisted of individuals that produced with nymph diapause and mainly distributed in the southern regions.

* *p* < 0.05.

** *p* < 0.01.

*** *p* < 0.001.

### 3.3 Divergence time estimation

**FIGURE 6:**
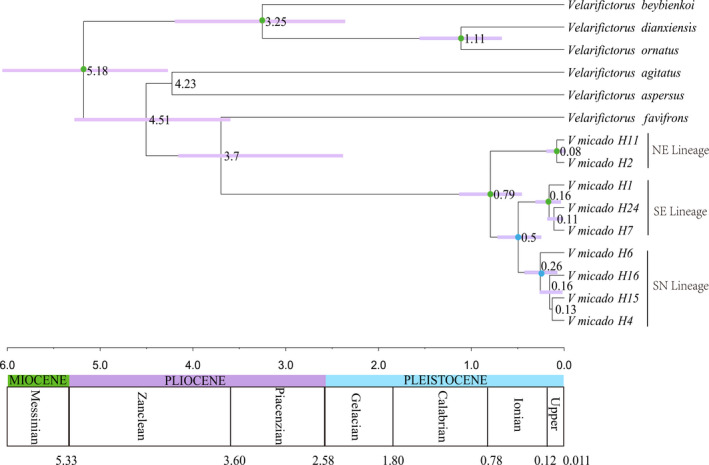
Time estimation of *Velarifictorus micado* based on COI constructed using BEAST. NE lineage represented the individuals that produced with egg diapause and mainly distributed in the northern regions, SE lineage represented the individuals with egg diapause and mainly distributed in the southern regions, and SN lineage represented individuals that produced with nymph diapause and mainly distributed in the southern regions. Purple bars represent the node age 95% credible intervals. The numbers of the nodes were the estimated node ages. Green circles and blue circles on nodes indicate posterior probability values over 95 and 90, respectively

The substitution models were selected under JModeltest v.2 (Darriba et al., 2012). The biggest Bayesian information criterion (BIC) was preferentially used to compare substitution models, and HKY+I+G model of sequence evolution was selected. The parameter estimates of ESS were more than 200 in Tracer v1.7.1. The chronogram reconstructed with BEAST was based on COI (Figure 6). The most recent common ancestor for all *V. micado* was dated at approximately 0.79 Ma (95% HPD: 1.13–0.46 Ma). The diversification time between SN and SE clades was 0.50 Ma (95% HPD: 0.71–0.25 Ma).

## 4 DISCUSSION

### 4.1 Life cycle and distribution

*Velarifictorus micado* has two ways to get through the winter, nymph diapause, and egg diapause (He & Takeda, 2013). Populations with egg diapause developed later than that with nymph diapause. Adults of egg diapause population were found from July to October (Shandong, Heilongjiang, Henan, Guizhou and Yunnan provinces), and nymphs were from July to August (Shandong and Yunnan provinces). Adults with nymph diapause appear earlier, and nymphs could also be found later, because the next generation hatched before winter. The individuals with nymph diapause, as we found, were adults in March (Hainan province), April (Guangxi province), June (Hubei and Zhejiang provinces), and July (Guizhou province). The nymph was found in the spring or the later autumn, such as March (Hainan province), April (Guangxi province), August (Guizhou province), September, and October (Zhejiang province) (Table 3).

As the previous research showed, there are seven main types of life histories in crickets, (1) continuous breeding without any diapause in environments without marked seasonal changes. (2) One generation in each year with egg diapause during winter or dry seasons. (3) One generation in each year with juvenile diapause during winter. (4) One generation in each 2 years with diapause in both egg and juvenile stage during winter. (5) One generation in each 2 years with diapause in both juvenile and adult stage during winter. (6) Two generations in each year with juvenile diapause during winter. (7) Two generations in each year with egg diapause during winter (Alexander, 1968). *V. micado* has two modes of (2) and (3). The different life history reduced hybridization between diapausing and nondiapausing populations (Hogan, 1967), and one species with two life cycles, rather than geographic isolation, might result in speciation at certain latitudes (Hogan, 1967; Masaki, 1983). However, there are two questions unresolved. Which life cycle is the ancestral type? How the new life cycles originate? Hogan (1966) believed that the ancestor would be nondiapausing. After introduced to temperate regions, their life history has changed (Hogan, 1966). Walker proposed two hypotheses. The first one was consistent with Hogan, and the second was that the residents transferred to south and turned to produce multiple generations each year with the warmer climate (Walker, 1980). Our results suggested the northern egg‐diapausing, the southern egg‐diapausing, and the southern nymph‐diapausing comprised a strongly supported monophyletic group, respectively. This was similar with previous studies that different life cycles of crickets comprised independent groups (Harrison & Bogdanowicz, 1995; Snyder et al., 2009). According to the biogeographic feature, the egg‐diapausing distributed in most regions of East Asia ranging from 24° to 46°N. The nymph‐diapausing distributed essentially in the south of Yangtze River in China, the northern limit reached to 32°N (Figure 2). As other researches showed, with the decrease of latitudes, the number of generations each year of *L. migratoria* (Orthoptera: Acrididae) increased in China (Chen, 1999; Guo et al., 1991; Tanaka & Zhu, 2008). With the increase of latitude, the diapause mode of *V. micado* changed from nymph to egg diapause. Our results suggested that there were both ancient and undifferentiated egg and nymph diapause haplotype in Yunnan, Guizhou, and Zhejiang provinces. However, the haplotype in Zhejiang was single. Thus, the egg diapause in southern regions might originated from the southwestern China (Yunnan and Guizhou) (Figure 5, Table 3 and Table 9); then, they colonized the new habitat gradually, rather than new life cycle produced in the same location. It is possible that the nymph‐diapausing was the ancient life cycle, and the cricket in southwestern China changed to get through winter as egg with the cooling of the Pleistocene climate. Our study also indicated that the life cycles of crickets appeared to be plastic when their habitats moved between different climate zones (Snyder et al., 2009).

**TABLE 9:** Haplotypes, neutrality tests, and nucleotide polymorphism of 34 populations based on CytB

Population	Hap (CytB)	Tajima's D	Fu' Fs	Hd	π
HLJ	H 13(11), H 14(1)	−1.14053	−0.47566	0.40500	0.00077
HNJY	H 12(14), H 17(1), H 33(1), H 34(1), H 35(1)	−1.85306**	−3.48596***		
HNPY	H 12(1)				
SDDY	H 1(1), H 12(7), H 15(1), H 18(1), H 19(1), H 20(1), H 21(1)	−1.02001	0.47559	0.73100	0.00934
SDTA	H 12(9), H 17(2), H 19(1), H 23(1), H 24(1), H 25(1)	−1.66013*	−3.61846***	0.64800	0.00135
AHHF	H 1, H 17				
ZJSX	H 1, H 12(4), H 16(2), H 17(7), H 44, H 45, H 46, H 47	−2.03200*	−0.89348	0.81700	0.0056
SHMH	H 12, H 17(2)				
JPSS	H 49				
HBXY	H 1(2), H 36(2)				
HBWH	H 2				
ZJTT	H 1(8), H 2(3), H 3, H 4, H 11,	−0.80896	−0.69299	0.65900	0.00252
ZJTM	H 1, H 12, H 15, H 22(2)				
ZJWYL	H 2(4), H 2(2)				
ZJDP	H 1, H 2(3), H 4(28), H 22, H 26, H 37, H 38(2), H 39, H 40, H 41(2)	−1.93793*	−5.751	0.53300	0.00178
SHBS	H 1(7), H 12(5), H 15(5), H 16, H 17(3), H 31	2.35748	6.53209	0.81000	0.01623
GZXY	H 4(7), H 28, H 29, H 30	−1.21975	−1.68362	0.61800	0.00201
GZGY	H 4				
GZRH	H 4				
GZZY	H 4				
ZJFY	H 4				
ZJBSZ	H 4(24)	0.00000	0.00000	0.00000	0.00000
YNKM	H 4(8), H 26(17), H 27	−0.01682	0.04503	0.49500	0.0009
YNMK	H 4				
GXPM	H 4(5), H 8 (2)				
GXSL	H 4(2), H 8				
GXJX	H 4(3), H 8(2), H 10(2)				
YNHK	H 4				
HNJFL	H 4., H 5, H 6(2)				
HNWZ	H 4				
HNXA	H 4(2), H 5				
HNCJ	H 4, H 5, H 7				
ZJGT	H 2(4)				
USAVA	H 47				

Note

Hap, haplotype distribution; Hd, haplotype diversity; π, nucleotide diversity.

* *p* < 0.05.

** *p* < 0.01.

*** *p* < 0.001.

The wide sampling in East Asia enabled us to find the distribution of two groups with different life cycles and compare differences between them. The Yangtze River is essentially a biogeographic boundary. The egg diapause populations (NE and SE) live in both the north and south of the Yangtze River. Based the COI, the NE lineage contained the populations distributed in north of the Yangtze River regions as well as those in Shanghai and Zhejiang. The individuals of SE lineage distributed in the south of the Yangtze River regions as well as those in Hubei, Jiangsu, and Shandong. The SN lineage included the populations distributed in the south of Yangtze River and Hubei (Figure 2). This phenomenon with two life histories bounded by the Yangtze River could be seen between *Teleogryllus emma* and *Teleogryllus occipitalis* (He et al., 2017; Lu et al., 2018). Wing dimorphism has been found in *V. micado*. The macropterous are capable of flying and the micropterous are flightless (Wu et al., 2014). However, there were only four macropterous individuals in our samples. It is possible that the migration ability of this cricket is weak. Obviously, there were at least two lineages in Jiangsu, Anhui, Shanghai, Zhejiang, and Yunnan provinces, so these are contact zones, which probably exists in other regions where has not be discovered due to the limited sampling time and size. According to the distribution of species, there is a broad faunal transition zone in the Quaternary between the Palearctic and Oriental realm in China (Zhang, 2002) and no strict biogeographic division (Norton et al., 2011). Although it is not strictly defined by the Yangtze River, our results were basically consistent with an suggestion of Wallace that the Yangtze River is the biogeographic boundary between the Oriental and Palearctic (Wallace, 1876). The sympatric coexistence of *V. micado* with different life cycles (Zhejiang, Guizhou, and Yunnan provinces) might result from the recent expansions of different populations, instead of new life cycle produced in the same location. The positive value of neutral test, the genetic differentiation indices (*F_ST_*), and the divergence time estimation provide reliable evidences to support that the differentiation among them has occurred for a long time (Figure 6, Table 2 and Table 5). The divergence between them is maintained in the absence of obvious environmental difference and barriers to gene flow, such as the same photoperiod, temperature, humidity,, and habitats, while the genomic underpinning of ecological speciation often appear to have been found to be the result of a long period of allopatry (Bernatchez & Dodson, 1990; Feder et al., 2003; Foote & Morin, 2015; Gray et al., 2006; Kuehne et al., 2007; Lucek et al., 2018).

### 4.2 The invasive origin of the USA

Our results confirmed that *V. micado* was transferred to America (Figure 2 and Figure 4) (Bowles, 2018). *V. micado* was firstly found in the USA in 1959 (Alexander & Walker, 1962), and then, they were widely distributed around eastern and southeastern America (Peck et al., 1992; Walker, 1977). The recent research indicated the cricket had dispersed both northwards and westwards (Bowles, 2018). Our results revealed that there were at least twice invasions from Asia, one from eastern China (Hap 7) and another from Japan (Hap 5). Hap 8 might be introduced from Japan. Although it have not found in Japan due to the small sampling size, Hap 8 was related to Hap 5. They both got through winter as egg (Figure 4 and Table 3). Their distribution range in the United States may continue to expand due to their adaptation to dry and cold areas, for example, NE lineage was widely distributed in the northeastern China. The similar latitude and climate made the species origin from the USA easy to settle down to China, such as *Corythucha ciliata, Homalodisca coagulata,* and *Anopheles quadrimaculatus* (Li et al., 2007; Wang et al., 2008; Zhang et al., 2008). Similarly, *V. micado* was easy to adapt to the environment of the USA. Limited to sampling size, it is unknown if there are nymph populations in the USA (these may disperse southern). Our results suggested that the mitochondrial fragment COI could be easily used to check the origin of *V. micado* for prevention of invasion. Although it has not been found that this cricket has destructive damage to human or nature, the impact to ecosystem area of the new colony is unknown.

### 4.3 Divergence time and major events

Haplotype network based on COI well supported that *V. micado* has diversified to three lineages (NE, SN and SE). Divergence time between northern lineage (NE) and southern lineages (SE and SN) was dated to 0.79 Ma (1.13–0.46 Ma) during the Middle Pleistocene Transition (MPT) due to the tendency of change toward cooling climate. A following divergence of southern lineages into two sublineage, SE lineage (populations overwintered in the egg stage and mainly distributed in southern region) and SN lineage (populations with nymph diapause and mainly distributed in southern region), occurred during ~0.50 Ma (0.71–0.25 Ma) due to the dry and cool climate (Figure 6). The climatic transformation of Asia occurred in 0.8 Ma was closely related to Mid‐Pleistocene Transition (1.2–0.7 Ma), which was called due to glacial–interglacial cycles from a 41 to 100 ka dominant frequency (Clark et al., 2006; Pisias & Moore, 1981; Raymo et al., 1997; Ruddiman et al., 1989), and the Kunhuang (Kunlun Huanghe) movement of 0.9 Ma BP (Li et al., 2001, 2004). During these global glaciers after the Kunhuang movement, glaciers developed gradually in various parts of the QTP (Wu et al., 1999; Yao et al., 2000; Zheng, 2000; Zhou et al., 2002). During MPT, the intensified aridification and the cooling climate have major impact on restructuring of vegetation and faunas in temperate northern East Asia. The steppe replaced the forest, some mammalian fauna extincted and new species produced due to the climate and related vegetation changes since MPT (Zhou et al., 2018). Subtropical zone reached at 42°N before the middle Pleistocene and retreated southwards after the mid‐Pleistocene (Liu & Ding, 1983). Many species immigrated southwards, including boreal, thermophilic, and humid‐preferring fauna. Species adapted to the new environment, while some remained and scattered in the locality during the Quaternary glaciation (Zhang, 2004). During the MPT, mid‐Pleistocene *Homo* (MPH) (Bae, 2010; Wu & Poirier, 1995) in East China adapted to the diverse and various climate, distributing from the temperate to subtropical zone (Guo et al., 2019; Kong et al., 2018). The climate shifts in MPT impose a strong influence on the diversification and distribution change of species. Thus, individuals distributed in northern and southern China diverged into two main lineages due to the cooling climate and intensified aridification. Besides, we observed that this cricket is common around the habitats of human, but not in the wild. It is possible that human behavior influenced the distribution of the cricket to some extent after the Mid‐Pleistocene.

The second differentiation occurred during ~0.50 Ma (0.71–0.25 Ma) between SE and SN lineages, when glaciers developed gradually in various parts of the QTP. The glaciation occurred at 0.73 Ma in Kunlun Mountain in northern QTP (Wu et al., 1999). The glaciation time in Yunshanping in southeastern QTP was 0.47–0.71 Ma (Yao et al., 2000; Zheng, 2000). The earliest glaciation time was dated to 0.46 Ma in Qilian Mountain in northeastern QTP (Zhou et al., 2002). Thus, glaciation and climatic shifts might trigger individuals in southern region diverging into two sublineages (SE and SN). Our results suggested that there were both undifferentiated and ancient haplotype with egg and nymph diapause in Yunnan, Guizhou, and Zhejiang provinces. However, the haplotype in Zhejiang was single. Thus, the SE lineage might originate from the southwestern China (Yunnan and Guizhou) (Figure 5, Table 3 and Table 9).

Our results indicated that lineage diversification of this cricket was driven by the climatic occasions after Mid‐Pleistocene, and the different life cycles played an important role in that. But it is unknown whether the change of life‐history strategy attributed to the climatic occasion after Mid‐Pleistocene. Studies in fishes suggested that life history is related to environmental factors (Marten et al., 2006; Papadopoulou et al., 2009), and influence the gene flow, and thus lineage diversification (Fluker et al., 2014; Tibbets & Dowling, 1996; Whiteley et al., 2004). We had two hypotheses about the evolution of life cycle. If the evolution time of life cycle was much earlier than that of lineages, the egg‐diapausing might be the ancient type, which was also supported by the Bayes tree (Figure 6). However, if the evolution time of life cycle was similar to that of lineages, it is possible that the nymph diapause turned to egg diapause twice due to cooler climate after Mid‐Pleistocene and diverged to three lineages. Namely, the nymph‐diapausing might be the ancient type. Individuals in northern regions turned to egg diapause resulted from the climatic variability, and the individuals with nymph diapause were filtered out under natural selection in northern regions.

It should be noted that there are two questions remain to be solved. First, the range of the sampling time and sampling region should be increased to further clarify the distribution of individuals with the other mode of life cycle. For example, it need to sample in Japan and America to clarify the origin of special haplotype (Hap 8) and whether there was nymph diapause. Second, the ancient type of *V.micado* is still indeterminate. Further research could study the ancient type of life cycle through the related genes.

## CONFLICT OF INTEREST

The authors declare no conflict of interest.

## AUTHOR CONTRIBUTIONS

**Baiqiu Wang:** Data curation (lead); formal analysis (lead); investigation (lead); writing – original draft (lead); writing – review and editing (lead). **Kai Li:** Funding acquisition (lead); project administration (lead); resources (lead); supervision (lead); writing – review and editing (lead). **Zhu‐Qing HE:** Funding acquisition (lead); investigation (equal); project administration (lead); supervision (lead).

## Data Availability

DNA sequences are depositing to GenBank under Accession nos, MT996525–MT997653.
